# Analysis on Adhesively-Bonded Joints of FRP-steel Composite Bridge under Combined Loading: Arcan Test Study and Numerical Modeling

**DOI:** 10.3390/polym8010018

**Published:** 2016-01-15

**Authors:** Xu Jiang, Xuhong Qiang, Henk Kolstein, Frans Bijlaard

**Affiliations:** 1 Department of Bridge Engineering, College of Civil Engineering, Tongji University, Shanghai 200092, China; jiangxu@tongji.edu.cn; 2 Department of Structural Engineering, College of Civil Engineering, Tongji University, Shanghai 200092, China; 3 Faculty of Civil Engineering and Geosciences, Delft University of Technology, Delft 2628CN, The Netherlands; M.H.Kolstein@tudelft.nl (H.K.); F.S.K.Bijlaard@tudelft.nl (F.B.)

**Keywords:** FRP deck, adhesively-bonded joint, combined loading, finite element analysis, failure criterion

## Abstract

The research presented in this paper is an experimental study and numerical analysis on mechanical behavior of the adhesively-bonded joint between FRP sandwich bridge deck and steel girder. Generally, there are three typical stress states in the adhesively-bonded joint: shear stress, tensile stress, and combination of both. To realize these stress states in the adhesively-bonded joint during tests, a specific loading device is developed with the capacity of providing six different loading angles, which are 0°(pure tension), 18°, 36°, 54°, 72° and 90°(pure shear). Failure modes of adhesively-bonded joints are investigated. It indicates that, for the pure shear loading, the failure mode is the cohesive failure (near the interface between the adhesive layer and the steel support) in the adhesive layer. For the pure tensile and combined loading conditions, the failure mode is the combination of fiber breaking, FRP delamination and interfacial adhesion failure between the FRP sandwich deck and the adhesive layer. The load-bearing capacities of adhesive joints under combined loading are much lower than those of the pure tensile and pure shear loading conditions. According to the test results of six angle loading conditions, a tensile/shear failure criterion of the adhesively-bonded joint is obtained. By using Finite Element (FE) modeling method, linear elastic simulations are performed to characterize the stress distribution throughout the adhesively-bonded joint.

## 1. Introduction

Fiber Reinforced Polymer (FRP) bridge decks are increasingly used in practice for rehabilitation of existing bridges and also for new bridge constructions, which are due to the remarkable advantages of FRP decks: lightweight of bridge superstructures, ease of installation, minimum traffic disturbance, large tolerance for environmental corrosion, long service life, as well as low maintenance cost. To be cost-effective, FRP decks are commonly supported by longitudinal main girders made of steel, prestressed concrete or FRP composites [1,2,3,4]. Between FRP decks and steel girders, adhesive bonding is usually employed as a preferable connection method. Compared with welding and bolted connections, it can reduce constructional time, save weight by eliminating fasteners, introduce more uniform load transfer and provide better long-term performance. Adhesively-bonded connections have been intensively investigated over the past 70 years. Most researches were related to the aerospace and aircraft engineering industry [5]. During the last two decades, the adhesive bonding technique was increasingly used in civil Engineering field, especially for repairing the deteriorated concrete components (beam and column) and structures using Carbon Fiber Reinforced Polymer (CFRP) sheets [6]. As FRP profiles are increasingly used in civil infrastructures, researches focused on the mechanical performance of FRP adhesively-bonded single-lap joints and double-lap joints were conducted [7,8,9,10,11]. These adhesive joints composed of pultruded Glass Fiber Reinforced Polymer (GFRP) composite profiles glued by epoxy adhesives. Parametric studies were conducted experimentally and numerically on the overlap length, adhesive layer thickness, adherend thickness and degree of chamfering of adherends. The results indicated that the combination of local through-thickness tensile (peeling) stress and shear stress was the most severe stress-state and usually initiated the failures in the adhesive fillet and in the outer fiber-mat layers of the adherends below the joint edges. A probabilistic strength prediction method on the adhesive joints was also investigated, considering the scale sensitivity by using Weibull statistical distribution [11].

Recently, for traditional concrete-steel composite bridges, adhesively-bonded connections started to get more attention to be utilized between concrete deck and steel girder [12,13,14,15,16,17]. The opportunity to substitute the traditional metallic connection (e.g., studs) with the adhesive bonding connection can be due to the advantages, including the reduction of construction time, continuity of force transmission and consequently a reduction in the risk of transversal cracks in the concrete, elimination of welding in connectors, more effective protection against steel girder corrosion and more possibility to use precast concrete for reducing the concrete shrinkage cracks [14]. Investigations on the adhesively-bonded connection between steel and concrete indicated that the adhesive bonding definitely enhanced the stiffness and strength of adhesive connections to levels almost as high as or higher than those of the traditional steel stud connection [12,13,16,17]. In Berthet *et al.*’s research [12], by employing push-out tests, the average measured shear stress of adhesive connections was found to be between 3.4 and 5.4 MPa, which was larger than the shear strength of concrete (3 MPa). The peeling stress was evident due to the additional bending moment, which influenced the ultimate load of the composite structures. Further researches were performed on the laboratory tests of 3–5 m span beams [13].The study showed that bonding concrete to steel resulted in less slip between the two components comparing with steel stud connections. The brittle and unforeseen failure mode of specimens may be explained by combined peeling and shear stress in the concrete. To better understand the influence of combined peeling and shear stress on the strength of concrete-steel adhesively-bonded joints, a further research was conducted [14]. Finally, the quadratic tensile-shear interaction failure criterion was established and validated by experimental steel-concrete composite beams, which was recommended for the design of steel-concrete bonded composite bridges.

For adhesively-bonded joints between the FRP bridge deck and the longitudinal steel girder, the technical background and researches have not been documented adequately in the literature. To fill in this gap, in this paper, the mechanical behavior of FRP-to-steel adhesively-bonded joints is experimentally and numerically investigated. A tensile-shear loading device is designed with the adaptability to provide the combination of tensile and shear loads in six different ratios. The quadratic failure criterion of the adhesively-bonded joint is obtained according to the test results. To characterize the stress distribution throughout the adhesively-bonded joint, Finite Element modeling method is employed.

## 2. Tensile-Shear Loading Device (Arcan Test Device)

**Figure 1:**
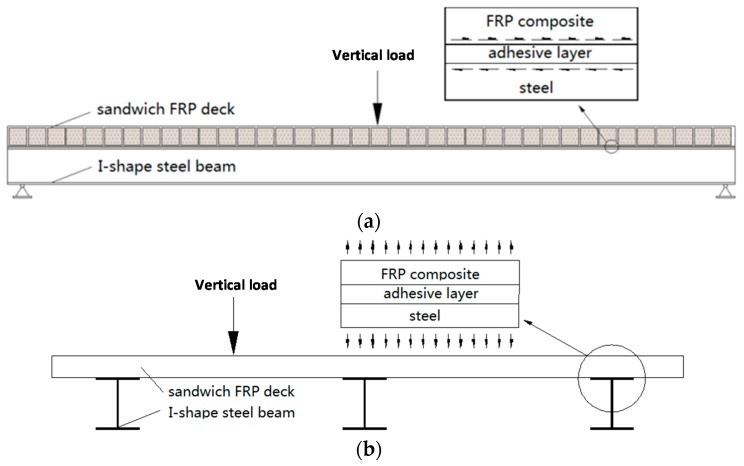
Typical stress states of the adhesive joint: (**a**) shear stress in the longitudinal direction; and (**b**) tensile stress in the transverse direction.

Generally, there are three typical stress states for the adhesive joint between FRP deck and steel girder:

1. Shear stress τ: due to the composite action between FRP deck and steel girder in the longitudinal direction of bridge, the deck and steel girder trend to bend together to carry the traffic load. Thus, the adhesive joints are under the shear stress condition to transfer the load from the FRP deck to the steel girder, as shown in Figure 1a;
2. Tensile stress σ: in the transverse direction of bridge, loading on other traffic lanes causes up-lift forces on adhesive joints, which results in the tensile stress (peeling stress), as shown in Figure 1b;
3. Combination of shear stress and tensile stress with different contribution ratios.

According to the above three stress states, a specific loading device is needed for providing tensile load, shear load and the combination of both simultaneously.

**Figure 2:**
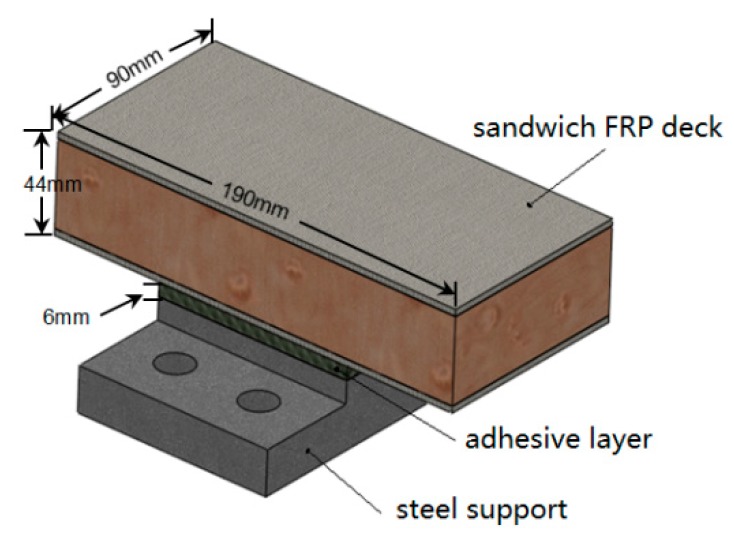
Adhesively-bonded joint.

**Figure 3:**
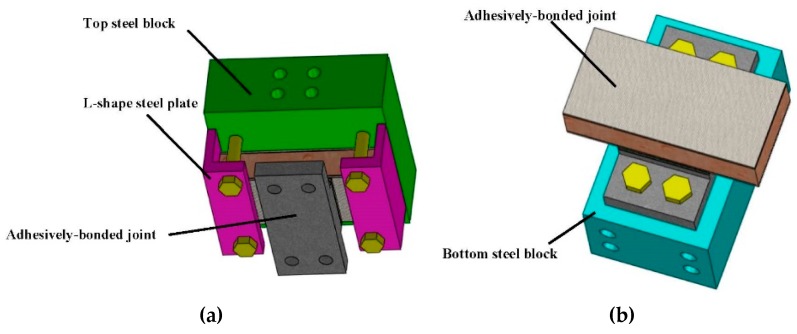
Joint fix configuration. (**a**) Deck fix configuration and (**b**) steel support fix configuration.

**Figure 4:**
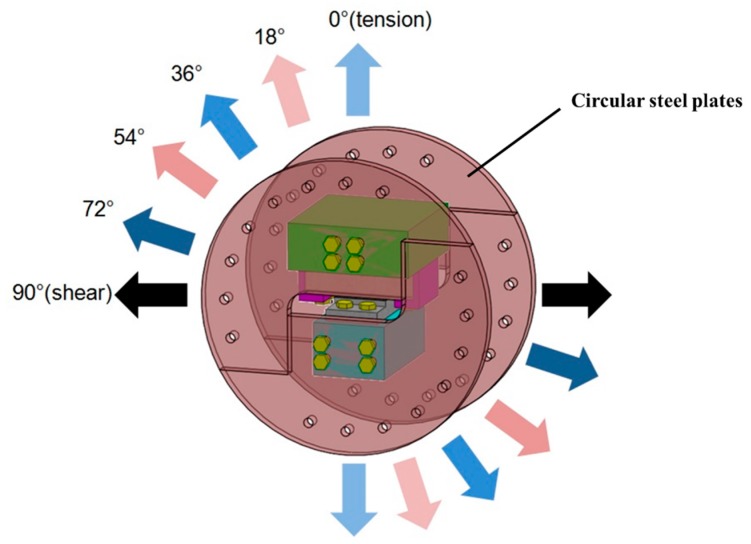
Tensile/shear loading device.

The adhesively-bonded joint between FRP deck and steel girder is extracted for experimental investigation as shown in Figure 2 190 mm × 90 mm sandwich bridge deck element is adhesively bonded to a convex shape steel support. The steel used herein is the commercial S355 grade steel. In the middle of sandwich deck element is the 38.1 mm Balsa SB150 (3A Composites [18]), a core material produced from certified kiln-dried balsa wood in the “end-grain” configuration. The surface layers are three layers of 0.94 mm EQX1200 (OCV Technical Fabrics [19]), which are the glass-fiber reinforced laminated polymer composites (54% glass content by weight). Properties of EQX1200 are shown in Table 1. The sandwich profiles are manufactured by resin vacuum infusion(Infra Composite B.V. [20]). The thickness of the 90 mm × 90 mm adhesive layer is 6 mm. Dimensions of adhesive joints are determined depending on the actual application of composite bridges as well as limitations of the loading equipment. In order to fix the adhesive joint to the loading system, other accessorial components are designed as shown in Figure 3. The steel support is drilled with 4 holes to connect to steel blocks via bolts. For the sandwich deck element, no hole is made, since the discontinued part in the FRP material causes more stress distribution distortion, which is not actual in the application of composite bridges. All the accessorial components are made of steel. Compared with the FRP composites and adhesive materials, the deformation of steel components can be neglected during tests, due to the relatively high stiffness of the steel material. To fix the sandwich deck part, it is designed to be fastened by two purple L-shape steel plates through four bolts to the top steel block, as shown in Figure 3a. The steel support is fastened directly through four bolts to the bottom steel block, as shown in Figure 3b. And then, the two steel blocks are fastened to circular steel plates, as shown in Figure 4. The circular steel plates are composed of two single pieces. Three bolts are employed to transfer the load uniformly. By loading the different angles of the circular steel plates, the proposed stress-state can be achieved in the adhesive joint, such as pure tension, pure shear and combinations of both. Correspondingly, six loading conditions can be realized through this loading system. Similar Arcan loading devices have been employed by other researches [21,22,23].

**Table 1:** Properties of EQX1200 (supplied by manufacturer [19]).

Product name	Total weight (g/m^2^)	Weight uniformity (g/m^2^)				
		Yarn roving				Knit yarn
		0°	+45°	90°	−45°	
EQX 1200	1,193	283	300	300	300	10

The sand blasting method is employed for the surface pretreatment on FRP laminates and steel supports. It starts from wiping specimen surfaces with acetone to eliminate any presence of oil used during the machining process. Then, the surfaces are abraded using a sand blasting machine to remove the impurities and oxide layer which can potentially exist. After that, re-degreasing and re-cleaning of the surface are done with acetone to remove the particles that can remain after sanding. Three replicated specimens are employed for each loading angle. Thus, in total, 18 pieces of specimens are prepared.

## 3. Experiment

**Figure 5:**
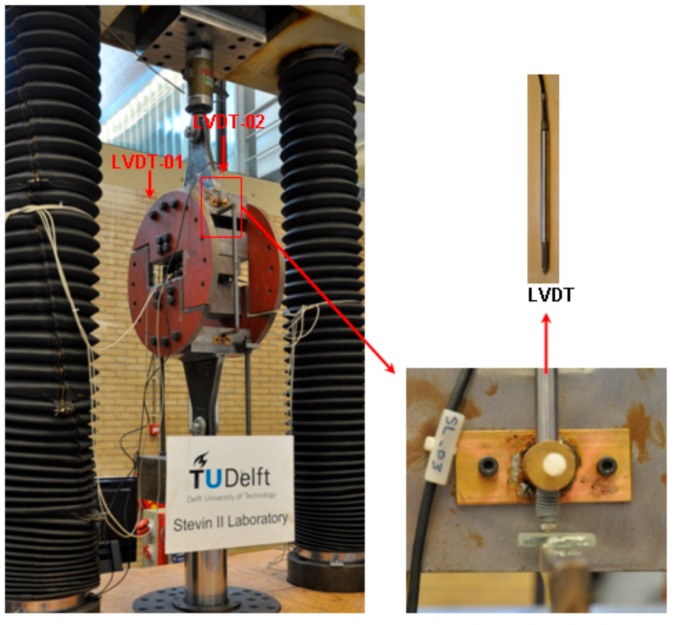
Test set-up.

The test set-up is shown in Figure 5. A SCHENCK Hydropuls test machine with loading capacity of 600 kN in tension is employed and controlled by the INSTRON 8400 controller, which can provide both load control and displacement control. The whole tensile/shear loading device is loaded by jacks through two hinged joints, which avoid the additional bending moment due to the eccentric loading from the specimen misalignment. Two LVDTs (linear variable differential transformer) are fixed on each side of the loading system, as shown in Figure 5, to measure the displacement between the top and bottom semi-circle loading device, for checking equal distribution of the load. The measure range of the LVDT is 0–10 mm. The quasi-static experiments of adhesively-bonded joints are displacement controlled by LVDTs at a rate of 0.001 mm/s. When the adhesive joint specimen is installed in the loading device, the four bolts connecting the L-shape steel profiles (see Figure 3a) are not fully fastened. A preload of 1kN is applied to ensure every loading component contact each other. In this way, the load can transfer smoothly from the loading device to the adhesive joint. After that, these four bolts are fully fastened and then the preload is unloaded. The tests start from 0 kN.

## 4. Results and Discussion

Table 2 lists failure loads of the adhesive joints under six load angles (0°, 18°, 36°, 54°, 72° and 90°). The lowest load-bearing capacity of the adhesive joints is obtained under the 18° angle loading condition, with an average failure load of 11.9 kN. The load-bearing capacity gradually increases from 11.9 to 23.27 kN as the loading angle increases from 18° to 72°. The largest deviation obtained is 34.0% under the shear loading condition, while the smallest deviation obtained is 5.55% under the 54° loading angle.

**Figure 6:**
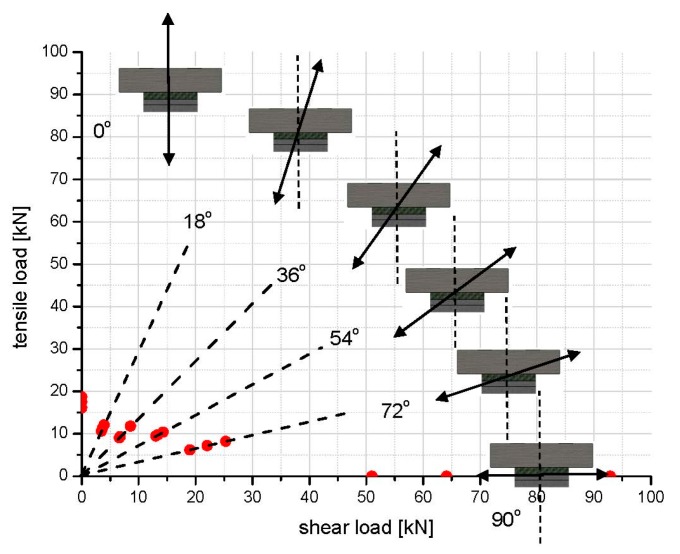
Failure loads of adhesive joints under six loading angles.

To easily recognize the load combination effects, the total failure load is vectorially separated into shear and tensile loads, with regard to the loading angle of each loading condition, as shown in Figure 6. The horizontal axis represents the shear load applied to the adhesive joint, while the vertical axis is the tensile load. It is apparent that, under the combination of tensile and shear loads, the load-bearing capacity of adhesive joints decreases, compared with that of pure tensile and pure shear loading conditions. From Table 2, the failure load of adhesive joints under the pure shear loading (69.3 kN) is considerably higher than that of the other loading conditions, due to the different failure modes, which are discussed in detail in the following sections. It is manifest that the failure load of an adhesive joint under different combined loading conditions is more sensitive to the percentage of tensile load vectorially separated from the resultant force. For the loading conditions of 18°, 36° and 54° angles, the vectorially separated tensile loads are close to each other, even though the deviations of failure loads are big. It means that, for a certain ratio limitation of the tensile and shear combination, the tensile load dominates the failure of adhesive joints more than the shear load. However, for the pure tensile loading condition (without any combination of shear load), the tensile failure load (17.43 kN) is much larger than the vectorially separated tensile loads (about 10kN) of the 18°, 36° and 54° angle loading conditions. It reconfirms that the combination of tensile and shear loads significantly degrades the load-bearing capacity of the adhesive joints.

**Table 2:** Ultimate failure loads of adhesive joints under six loading conditions.

Loading angle	Failure load (MPa)			Average	Deviation
	Specimen01	Specimen02	Specimen03		
Pure tensile	17.53	16.05	18.72	17.43	7.94%
18° angle	12.7	11.2	11.8	11.9	6.72%
36° angle	11.2	14.6	11.8	12.53	16.5%
54° angle	16.5	17.7	16.1	16.77	5.55%
72° angle	26.6	20.0	23.2	23.27	14.3%
Pure shear	51.0	92.9	64.1	69.3	34.0%

**Figure 7:**
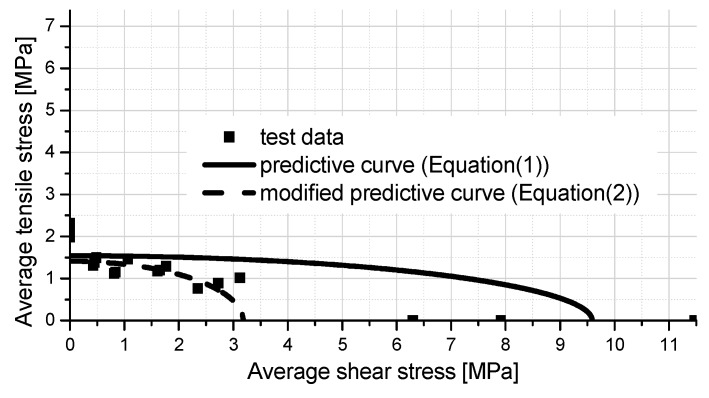
Shear–tensile failure criterion for the adhesively-bonded joint.

To investigate the failure criterion of adhesive joints, the value of average stress is considered, which is obtained using the tensile/shear load divided by the adhesive bonding area. An ellipsoid function is employed for curve fitting by the least square method, which was successfully used to establish the failure criteria of the concrete-steel adhesive joint under combined shear and peeling stresses [14]. Firstly, considering all the test results of six loading conditions, the predictive curve Equation (1) is obtained and indicated as the solid line in Figure 7. It can be found that the agreement between the test results and the predictive equation is not very well. All the test data of four combined loading conditions are below the predictive curve, which implies that the predictive equation is not conservative for the design of these adhesive joints. To solve this issue, only the test results of four combinations of shear and tensile loads are selected to perform as the basic curve fitting data. The modified predictive equation is expressed by Equation (2), and indicated as the dashed line in Figure 7. It can be found that the modified predictive equation is more conservative and practical, since in the real application of an adhesive joint between FRP decks and steel girders, the joint mainly carries the combined loading of tension and shear, but not only the pure tension or pure shear load.

Predictive curve:

(1) $${\left(\frac{\sigma }{1.54}\right)}^{2}+{\left(\frac{\tau }{9.59}\right)}^{2}=1$$

Modified predictive curve:

(2) $${\left(\frac{\sigma }{1.41}\right)}^{2}+{\left(\frac{\tau }{3.18}\right)}^{2}=1$$

**Figure 8:**
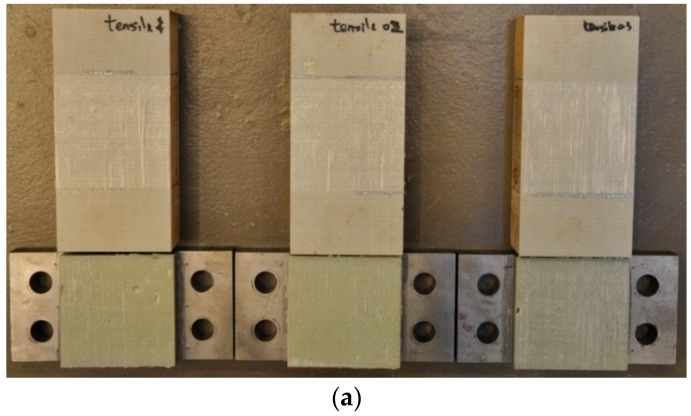

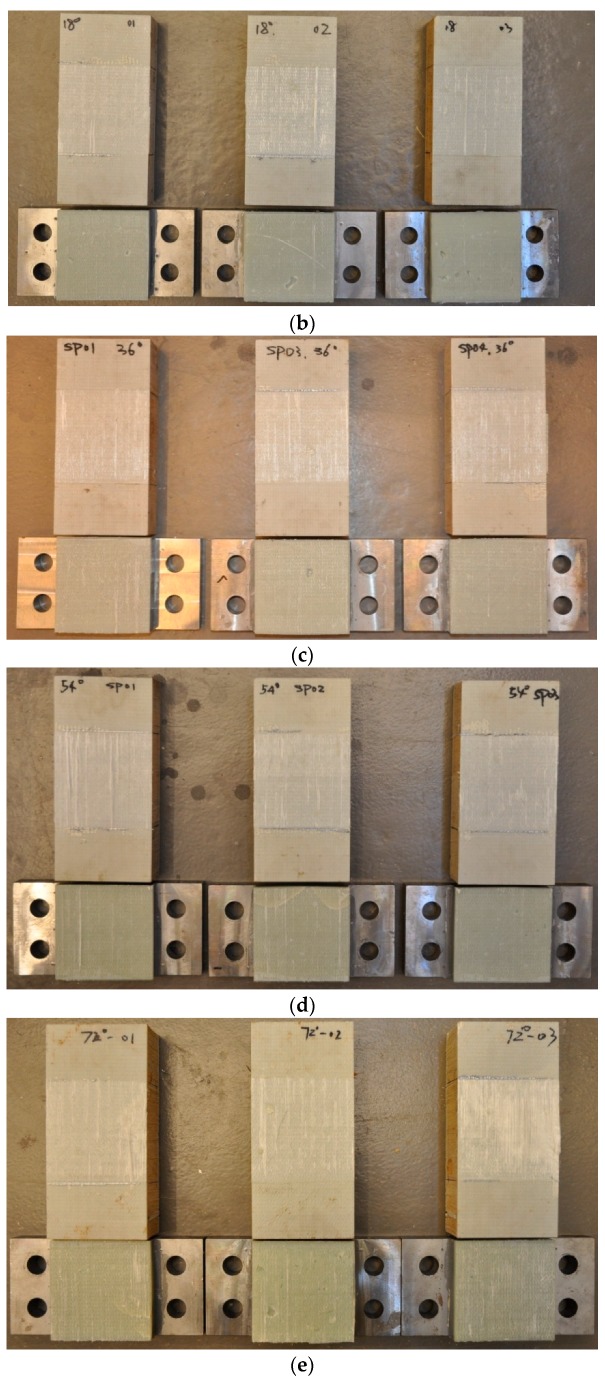

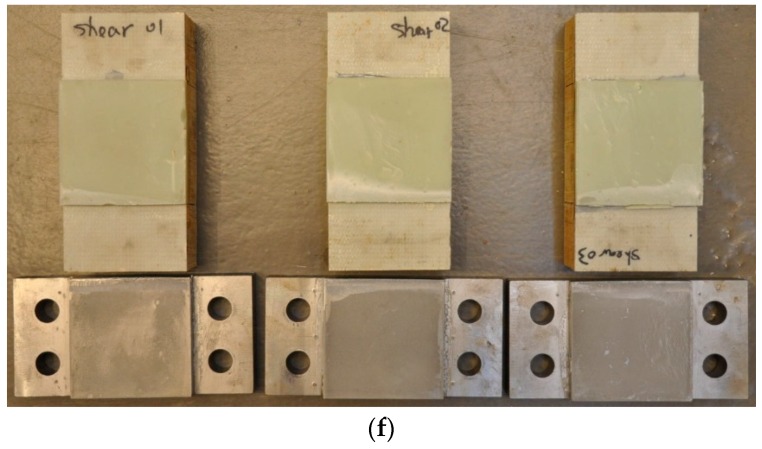
Failure modes of adhesive joints under six loading angles:(**a**)pure tension; (**b**) 18° angle; (**c**) 36° angle; (**d**) 54° angle; (**e**) 72° angle; and (**f**) pure shear.

**Figure 9:**
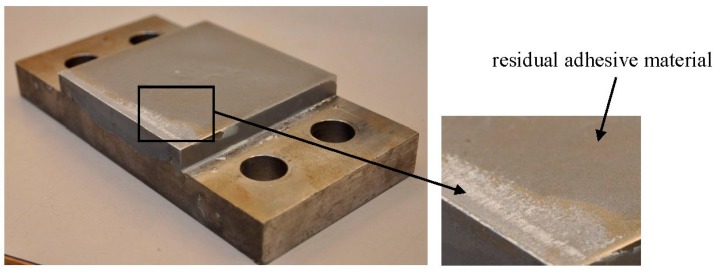
Cohesive failure of the adhesive joint under the pure shear loading condition.

The failure modes of all the test specimens are shown in Figure 8. For adhesive joints under the shear loading, the ultimate failure of the adhesive joints always occurs in a brittle and sudden manner. The failure mode is the cohesive fracture (near the interface between adhesive layer and steel support) in the adhesive layer, as shown in Figure 9. It is visible that some residual adhesive material is left on the surface of the steel support. It can be predicted from the fracture plane (see Figure 9), cracks initiate at the edge of the adhesive bonding area and steadily propagate towards the middle of the adhesive layer. The stress re-distribution occurs throughout the bonding area and continuously carries the load. When the rest adhesive bonding area is too limited to carry the load, the cracks rapidly develop through the whole section and the adhesive joint immediately loses its load-bearing capacity.

**Figure 10:**
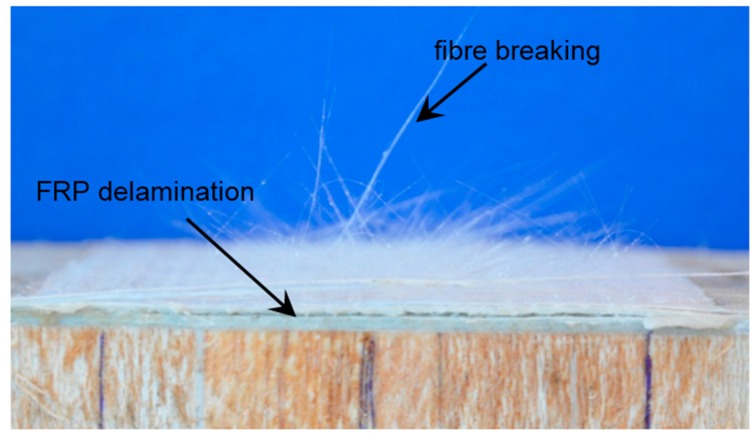
FRP delamination and fiber breaking in the FRP sandwich deck.

For the other five angle loading conditions, all the fracture planes are through the interface between the FRP sandwich deck and the adhesive layer. Some areas of fiber breaking or FRP delamination are evident from the view of the FRP laminate failure surface, as shown in Figure 10. But these areas do not fully cover the adhesively-bonded area. This failure mode can be defined as the combination of fiber breaking, FRP delamination and interfacial adhesion failure between the FRP sandwich deck and the adhesive layer. Furthermore, multiple failure modes under the pure shear loading and the other five loading conditions indicate that the shear load-bearing capacity of the adhesive layer is much lower than that of adhesive-steel and adhesive-FRP interfaces and the failure of the interface between the FRP sandwich deck and adhesive layer is dominated by the tensile load.

**Figure 11:**
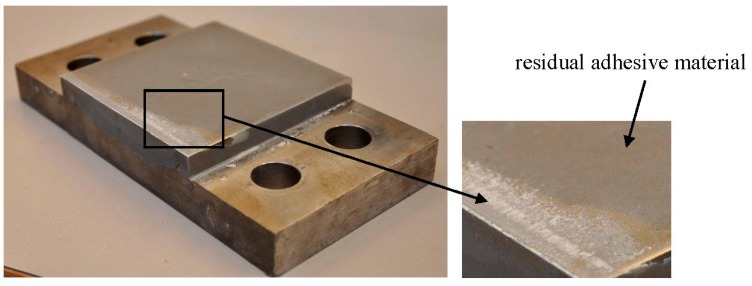
Cracks in the adhesive layer of the 72°-01 specimen under the 72° angle loading condition.

It is worthwhile mentioning that, for the 72°-angle loading condition, there are some cracks observed in the adhesive layer of the 72°-01 specimen and propagate through the interface between the adhesive layer and the steel support, as illustrated in Figure 11. It indicates that the failure plane almost switches to the interface between the adhesive layer and the steel support, which occurred for the adhesive joints under the pure shear loading condition. This phenomenon suggests that the upper and lower interfaces between the adhesive layer and the FRP laminate or the steel support almost achieve the failure homogenously. However, for the other two specimens under the 72° loading angle, cracks in the adhesive layer are not visible. Instead, a large portion of FRP delamination or fiber breaking area is observed. Although all the specimens were prepared and cured in the same condition, multiple failure modes under the 72° angle loading condition are obtained. This phenomenon is attributed to the different bonding qualities of specimens, which could be induced by the non-uniform distribution of voids in the adhesive layer or the different interfacial adhesion strengths between the FRP laminate and the adhesive layer. It indicates that the controllable adhesive bonding technique is essential to guarantee the reliable mechanical performance of the adhesive joints.

## 5. FE Analysis

### 5.1. FE Model

**Figure 12:**
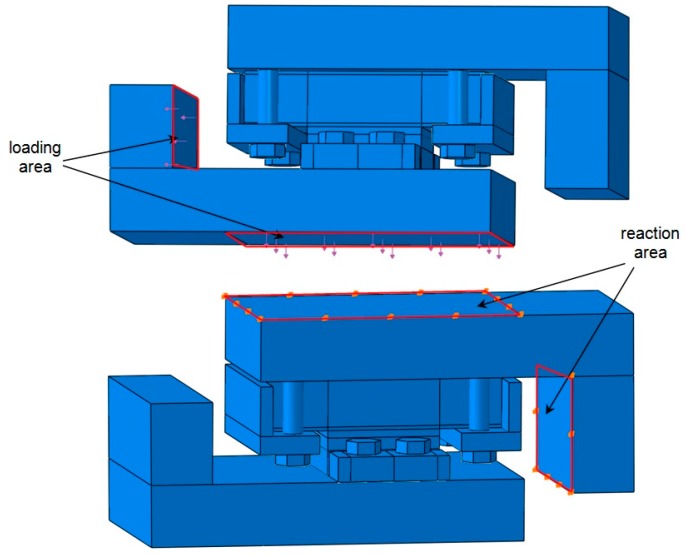
Loading and boundary condition of FE model.

**Figure 13:**
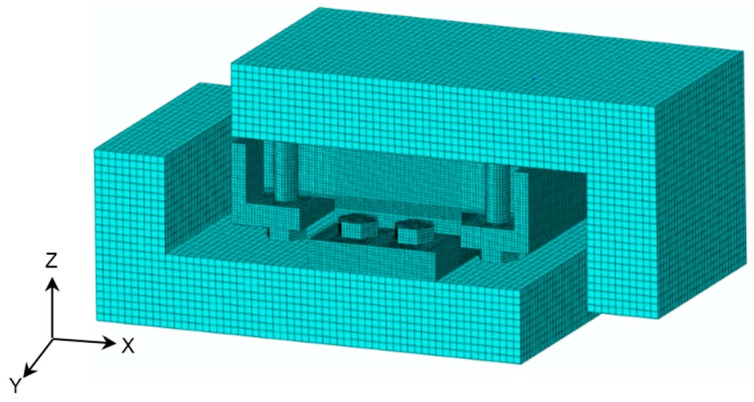
FE model.

**Figure 14:**
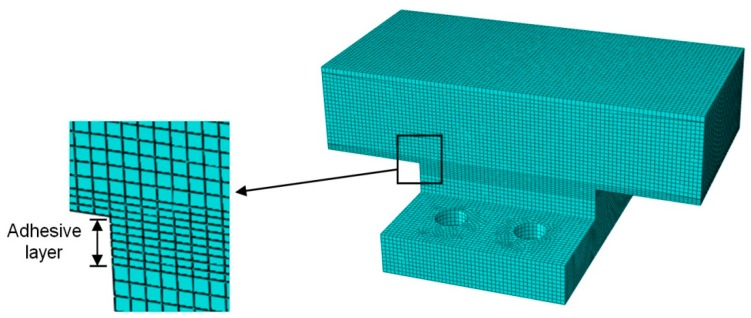
Mesh configuration of adhesive joint.

The FE model of adhesive joints under the combination of tensile and shear loads is developed using ABAQUS 6.8 software (Dassault Systèmes Americas Corp., Waltham, MA, USA). In order to save computational time, the FE model is simplified, as shown in Figure 12. The shear and tensile loads are applied via the surface tractions on the loading area respectively, while all the degrees of freedom are restricted for the reaction area. The center of shear and tensile loading area is exactly through the centroid of the adhesive layer, in such a way to confirm that the resultant force is also through the centroid of the adhesive layer and no additional bending moment is involved. Subsequently, four loading angles can be realized by varying the ratio between shear load and tensile load with specific tangent values. Depending on the investigations of mesh dependence of an FE model under the shear loading condition [24], the FE model with the 1.50 mm mesh scale and six-layer discretization through the thickness of the adhesive layer is preferable to achieve reasonable accuracy as well as to save computational time. Thus, this mesh configuration is employed for FE analysis in this research, as shown in Figure 13 and Figure 14. All the elements used are C3D8R, which are eight-node brick elements with reduced integration. No geometric non-linearity or elastic-plastic material properties are involved in the FE analysis. The input of material properties is listed in Table 3, which are supplied by the manufactures (3A Composites [18], OCV Technical Fabrics [19] and Infra Composites B.V. [20]) and the three-point bending test results of FRP laminates [25]. The lacking properties of balsa wood are referred to the Wood Handbook [26], and the material properties of steel are referred to the Chinese Design Code for Steel Structures [27]. The resultant force of 15 kN (vectorial combination of tensile and shear loads) is applied on the FE model. The linear FE results can be amplified by any ratio in order to be comparable with test results.

**Table 3:** Mechanical properties of materials for FE model.

Property	FRP Laminates
Elastic modulus 11 (MPa)	16,609
Elastic modulus 22 (MPa)	16,609
Elastic modulus 33 (MPa)	11,000
Poisson ratio 12	0.33
Poisson ratio 23	0.3
Poisson ratio 13	0.18
Shear modulus 12 (MPa)	6,986
Shear modulus 23 (MPa)	1,200
Shear modulus 31 (MPa)	1,200
**Adhesive**	
Elastic modulus (MPa)	3,400
Poisson ratio	0.37
**Core material**	
Elastic modulus 11 (MPa)	86
Elastic modulus 22 (MPa)	265
Elastic modulus 33 (MPa)	5,759
Poisson ratio 12	0.23
Poisson ratio 23	0.23
Poisson ratio 13	0.49
Shear modulus 12 (MPa)	29
Shear modulus 13 (MPa)	213
Shear modulus 23 (MPa)	309
**Steel**	
Elastic modulus (MPa)	206,000
Poisson ratio	0.3

### 5.2. FE Results and Discussion

**Figure 15:**
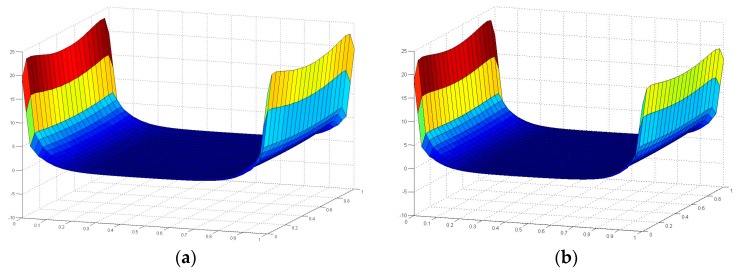

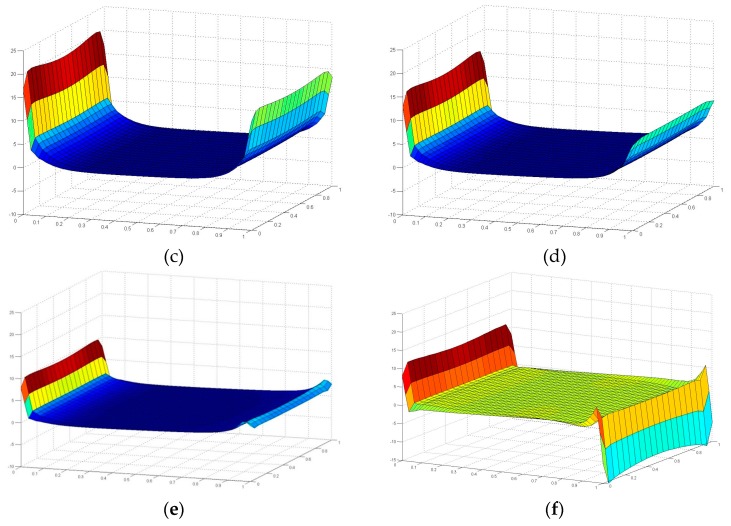
Contour maps of tensile stress on the interface between FRP laminates and adhesive layer under six loading conditions: (**a**) 0° angle loading (tensile); (**b**) 18° angle loading; (**c**) 36° angle loading; (**d**) 54° angle loading; (**e**) 72° angle loading; and (**f**) 90° angle loading (shear).

**Figure 16:**
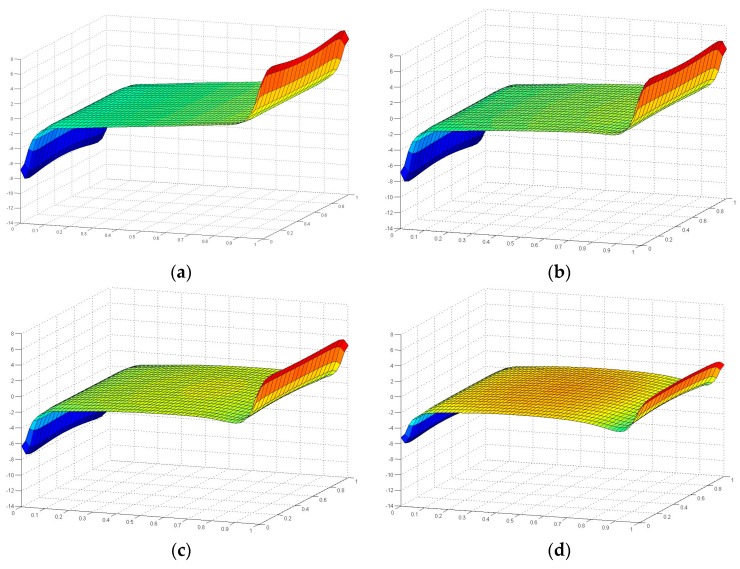

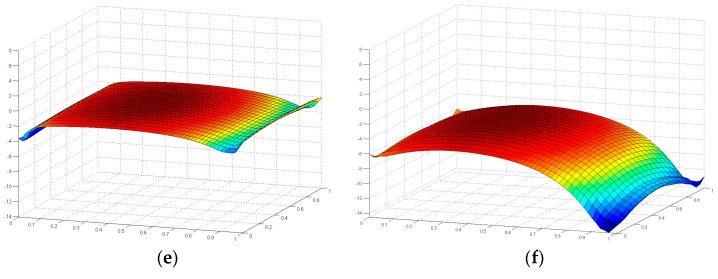
Contour maps of shear stress on the interface between FRP laminates and adhesive layer under six loading conditions:(**a**) 0° angle loading (tensile); (**b**) 18° angle loading; (**c**) 36° angle loading; (**d**) 54° angle loading; (**e**) 72° angle loading; and (**f**) 90° angle loading (shear).

**Figure 17:**
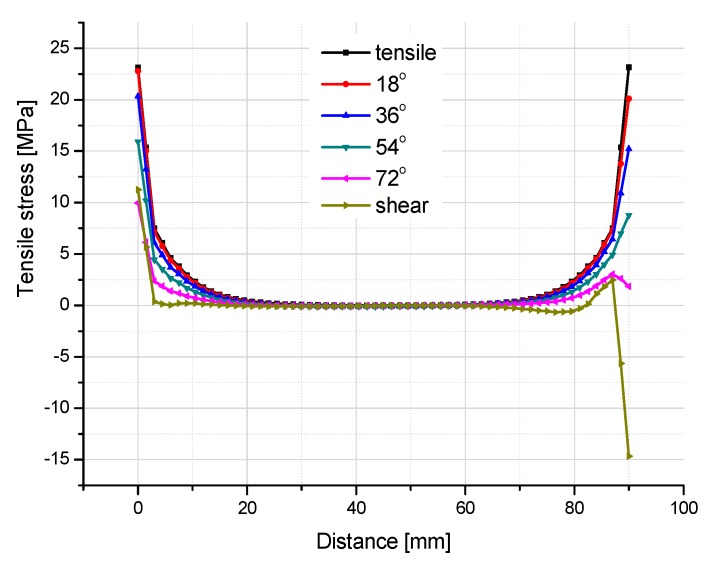
Tensile stress distribution in the longitudinal path at the location 9mm away from the end of the interface.

**Figure 18:**
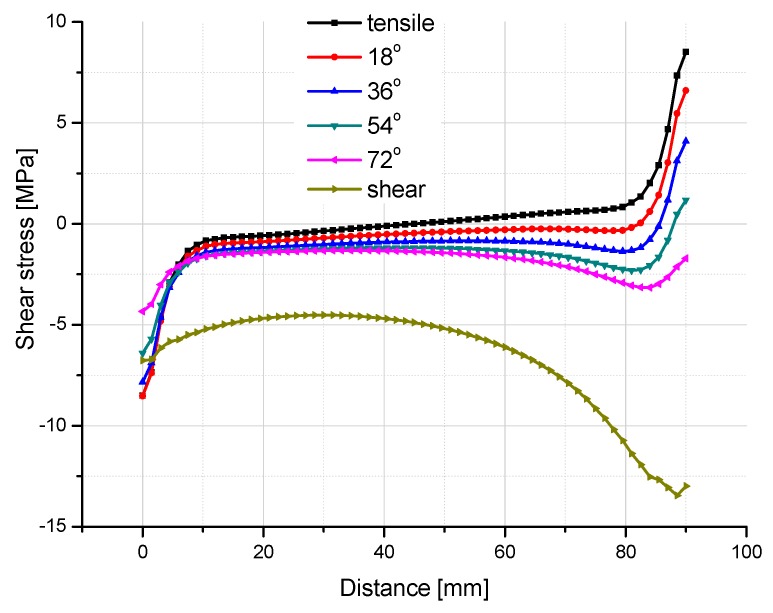
Shear stress distribution in the longitudinal path at the location 9mm away from the end of the interface.

Figure 15 and Figure 16 illustrate the contour map of tensile and shear stress on the interface between the FRP laminates and the adhesive layer under six loading angles, where the failure plane locates. For all the six angle loading conditions, in the transverse direction (the *Y* axis as indicated in Figure 13), tensile and shear stresses distribute more uniformly. The maximum absolute stress values are not located exactly at the ends of the interface, but at 9 mm distance from the end. To further investigate the stress distribution in the longitudinal direction (the *X* axis as indicated in Figure 13), the tensile and shear stresses are extracted through the longitudinal path at the location of 9mm distance from the end, as shown in Figure 17 and Figure 18.

From Figure 17, it can be found that for all six loading conditions the tensile stress is approximately zero in the central part of the interface in the longitudinal direction (the *X* axis as illustrated in Figure 13). However, in the vicinity of adhesive layer ends, the tensile stress concentration is evident, where the cracks can initiate and develop. As the loading angle rotates from 0° (tensile) to 90° (shear), the tensile stress singularity decreases at both ends of the interface, since the vectorially separated tensile load applied on the FE model of adhesive joint decreases. For the right end (shear load applied side), the extent of tensile stress decreasing is more significant than that on the left side, which introduces an asymmetrical tensile stress state throughout the longitudinal path. Subsequently, the tensile stress singularity at the right end drops to the negative zone for the shear loading condition. 

Figure 18 illustrates the shear stress distribution along the longitudinal path. The tensile and shear loading conditions achieve the two extreme shear stress states. The shear stress distributions of the other four angle loading conditions regularly transit from the tensile loading condition to the shear loading condition. In the central part of the interface, the shear stress values of four combined loading angles are not zero anymore. The absolute values are increasing with the vectorially separated shear load which increases from the 18° angle loading to the 72° angle loading. Without any doubt, the absolute value of shear stress achieves the maximum (13.5 MPa) for the shear loading condition (90° angle), which is many times higher than that under other loading conditions. This could be the reason that the failure plane of the adhesive joint under the pure shear load switched to the cohesive failure in the adhesive layer but not the fiber breaking or FRP delamination in the FRP laminates. For the right edge (the side where shear load applied), the variation of the shear stress state is more evident than that on the left side, since the shear stress at the load directly forced side is more sensitively influenced. To the contrary, the shear stress distribution at the left side does not vary too much for the six loading conditions. The three curves of 0° (tensile), 18° and 36° loading conditions almost cover each other.

## 6. Conclusions

From the experimental and numerical investigation on mechanical behavior of adhesive joints under six angle loading conditions, some conclusions can be drawn as follows:

(1) The load-bearing capacities of adhesive joints under combined loading are much lower than those of the pure tensile and pure shear loading conditions.
(2) According to failure loads of adhesive joints under the four combined loading conditions, the modified failure criterion of the adhesive joint is addressed by using aquadratic ellipsoid function, which is conservative and practical for the real application of an adhesive joint between FRP decks and steel girders in bridges.
(3) For adhesive joints under the shear loading condition, the failure mode is the cohesive failure (near the interface between the adhesive layer and the steel support) in the adhesive layer. However, for adhesive joints under the tensile loading and four combined loading conditions, the fracture plane is through the interface between the FRP sandwich deck element and the adhesive layer, combining with fiber breaking or FRP delamination in the FRP laminates.
(4) The controllable adhesive bonding technique is essential to guarantee the reliable mechanical performance of adhesive joints.
(5) The stress states on the interface between the FRP laminate and the adhesive layer under six loading angles are characterized using Finite Element modeling method, which gives a brief indication on crack initiation location and failure mode of the tested adhesive joints.

## References

[1] Knippers J., Pelke E., Gabler M., Berger D. (2010). Bridges with glass fibre-reinforced polymer decks: The road bridge in friedberg. Struct. Eng. Int..

[2] Sebastian W.M., Ross J., Keller T., Luke S. (2012). Load response due to local and global indeterminacies of FRP-deck bridges. Compos. Eng..

[3] Luke S., Canning L., Collins S., Knudsen E., Brown P., Taljsten B., Olofsson I. (2002). Advanced composite bridge decking system—project ASSET. Struct. Eng. Int..

[4] Keller T., Rothe J., de Castro J., Osei-Antwi M. (2014). GFRP-balsa sandwich bridge deck: Concept, design, and experimental validation. J. Compos. Construct..

[5] Adams R.D. (2005). Adhesive Bonding: Science, Technology And Applications.

[6] Hollaway L.C. (2010). A review of the present and future utilisation of FRP composites in the civil infrastructure with reference to their important in-service properties. Construct. Build. Mater..

[7] Keller T., Vallee T. (2005). Adhesively bonded lap joints from pultruded GFRP profiles. Part I: stress-strain analysis and failure modes. Compos. Eng..

[8] Keller T., Vallee T. (2005). Adhesively bonded lap joints from pultruded GFRP profiles. Part II: Joint strength prediction. Compos. Eng..

[9] Vallee T., Keller T. (2006). Adhesively bonded lap joints from pultruded GFRP profiles. Part III: Effects of chamfers. Compos. Eng..

[10] Vallee T., Correia J.R., Keller T. (2006). Probabilistic strength prediction for double lap joints composed of pultruded GFRP profiles part I: Experimental and numerical investigations. Compos. Sci. Technol..

[11] Vallee T., Correia J.R., Keller T. (2006). Probabilistic strength prediction for double lap joints composed of pultruded GFRP profiles—Part II: Strength prediction. Compos. Sci. Technol..

[12] Berthet J.F., Yurtdas I., Delmas Y., Li A. (2011). Evaluation of the adhesion resistance between steel and concrete by push out test. Int. J. Adhes. Adhes..

[13] Bouazaoui L., Perrenot G., Delmas Y., Li A. (2007). Experimental study of bonded steel concrete composite structures. J. Constr. Steel Res..

[14] Larbi A.S., Ferrier E., Hamelin P. (2009). Concrete to steel lap joint failure criteria under combined shear and peeling stress. J. Construct. Steel Res..

[15] Jurkiewiez B., Meaud C., Michel L. (2011). Non linear behaviour of steel-concrete epoxy bonded composite beams. J. Construct. Steel Res..

[16] Bouazaoui L., Jurkiewiez B., Delmas Y., Li A. (2008). Static behaviour of a full-scale steel-concrete beam with epoxy-bonding connection. Eng. Struct..

[17] Meaud C., Jurkiewiez B., Ferrier E. (2014). Steel-concrete bonding connection: An experimental study and non-linear finite element analysis. Int. J. Adhesion Adhes..

[18] 3A Composites product data sheet. http://www.3acomposites.com/.

[19] Nauticexpo Product Data Sheet. http://pdf.nauticexpo.com/pdf/vetrotex-ocv-reinforcements/quadraxial-fabrics/27891–13674-_2.html.

[20] Composite bridge decks. http://www.infracomposites.com.

[21] Créac'hcadec R., Sohier L., Cellard C., Gineste B. (2015). A stress concentration-free bonded arcan tensile compression shear test specimen for the evaluation of adhesive mechanical response. Int. J. Adhes. Adhes..

[22] El-Hajjar R., Haj-Ali R. (2004). In-plane shear testing of thick-section pultruded FRP composites using a modified Arcan fixture. Compos. Eng..

[23] Dos Santos D.J., Batalha G.F., Polimeros-Ciencia E. (2014). Failure criterion for adhesively bonded joints using Arcan’s experimental method. Tecnologia.

[24] Jiang X., Kolstein M.H., Bijlaard F.S.K. (2014). Experimental and numerical study on mechanical behavior of an adhesively-bonded joint of FRP-steel composite bridge under shear loading. Compos. Struct..

[25] Qiang X., Jiang X., Kolstein H., Bijlaard F.S.K. (2015). Mechanical degradation on flexural property of glass-fiber-reinforced polymer laminates under hot/wet environment. Compos. Eng..

[26] Wood Handbook, Wood as an Engineering Material. http://www.woodweb.com/Resources/wood_eng_handbook/wood_handbook_fpl_2010.pdf.

[27] (2003). GB50017-2003 Code for Design of Steel Structures.

